# Harnessing the power of microbial fuel cells as pioneering green technology: advancing sustainable energy and wastewater treatment through innovative nanotechnology

**DOI:** 10.1007/s00449-024-03115-z

**Published:** 2025-01-04

**Authors:** Hadeer E. Ali, Bahaa A. Hemdan, Mehrez E. El-Naggar, Mohamed Azab El-Liethy, Dipak A. Jadhav, Hoda H. El-Hendawy, M. Ali, Gamila E. El-Taweel

**Affiliations:** 1 Water Pollution Research Department, Environment and Climate Change Research Institute, National Research Centre, 33 El-Bohouth St., Dokki, 12622 Giza Egypt; 2 Pre-Treatment and Finishing of Cellulosic Fabric Department, Textile Research and Technology Institute, National Research Centre, 33 EL-Bohouth St., Dokki, 12622 Giza Egypt; 3 Department of Environmental Engineering, College of Ocean Science and Engineering, Korea Maritime and Ocean University, 727 Taejong-Ro, Yeongdo-Gu, Busan, 49112 Republic of Korea; 4 Botany and Microbiology Department, Faculty of Science, Helwan University, Cairo, Egypt; 5 Physics Department, Faculty of Science, Helwan University, Helwan, Cairo, Egypt

**Keywords:** Water scarcity, Energy crisis, Microbial fuel cells, Anode modifications, Wastewater treatment, Energy production

## Abstract

The purpose of this review is to gain attention about intro the advanced and green technology that has dual action for both clean wastewater and produce energy. Water scarcity and the continuous energy crisis have arisen as major worldwide concerns, requiring the creation of ecologically friendly and sustainable energy alternatives. The rapid exhaustion of fossil resources needs the development of alternative energy sources that reduce carbon emissions while maintaining ecological balance. Microbial fuel cells (MFCs) provide a viable option by producing power from the oxidation of organic and biodegradable chemicals using microorganisms as natural catalysts. This technology has sparked widespread attention due to its combined potential to cleanse wastewater and recover energy. The review presents a complete examination of current advances in MFCs technology, with a focus on the crucial role of anode materials in improving their performance. Moreover, different anode materials and their nanoscale modifications are being studied to boost MFC efficiency. This current review also focused on the effects of surface modifications and different anode compositions on power generation and system stability. It also investigates the electrochemical principles behind these enhancements, providing insights into the economic potential of MFCs. MFCs provide a long-term solution to energy and environmental issues by addressing both wastewater treatment and energy production.

## Introduction

Two essential components that support comfort and societal advancement are energy and water [1]. The widespread usage of fossil fuels during the past century has been crucial to the advancement of industrial and commercial development [2]. Due to environmental pollution, population growth, depletion of natural resources, and global warming, humanity has recently faced both an energy and water shortage [3]. A large amount of organic carbon molecules and thermal and chemical energy can be found in contaminated water; these chemical or organic carbons were utilized in synthesizing products with added value [4]. Purification is the main objective of conventional wastewater treatment systems instead of recovering resources [5]. The most popular biologic method of treatment is activated sludge, however membrane bioreactors and sequential batch reactors are also options [6]. Conventional wastewater treatment and resource recovery systems are expensive and energy-intensive due to their high operating and maintenance methods [7]. To address resource scarcity and environmental protection issues, there is significant interest in enhancing environmentally friendly methods for wastewater treatment and energy production [8]. Energy, alongside water, is important for the survival of civilization. Moreover, our need for a consistent power source heightens as civilization advances. The use of energy accounts for 60% of greenhouse gas emissions [9]. Energy is commonly considered a non-destructible system, but it is possible to transfer energy from one type of energy to another and between systems [10]. Expensive cost of maintaining energy generation, the lack of clean energy sources, accidents and natural catastrophes (which are increasing in frequency as a result of climate change), wars, and inadequate energy storage are additional factors leading to the severity of the crisis [11]. MFCs can be applied to treat wastewater in a more affordable and ecologically friendly manner to overcome this inefficiency [12]. In 1911, British botanist Potter [13] came up with a suggestion of employing bacteria that oxidize organic compounds to generate electricity. Since then, MFCs have gotten a great attention from researchers as a potentially viable energy generation solution. By consumption of organic contaminants found in wastewater, MFCs not only produce energy but also treat water [14]. The academic community has shown lots of interest in this concept during the past 10 years [15, 16]. When it was established in 1999 that bacteria could transmit electrons externally to electrodes, the idea that Potter had found in 1911 was finally understood [13]. The development of this technology has made it useful for both power generation and wastewater treatment [17]. The microbial inoculum, the electrode material, the chemical substrate in the fuel, and the proton exchange membrane (PEM) are the main elements that could affect MFC performance. The performance and, thus, the commercialization potential of MFCs are greatly influenced by the electrode material [18]. Numerous studies have demonstrated that the anode material, which makes it easier for exoelectrogens to connect, usually restricts the power output of MFCs [19]. Excellent mechanical strength and toughness, chemical stability and anticorrosion, biocompatibility, a large specific surface area, strong conductivity, and relatively low resistance are all characteristics that should be present in the best material [20]. The term “traditional anode materials” refers to both metallic (such as sheet copper and stainless steel) and carbonaceous (such as flake graphite, carbon paper, carbon cloth, carbon brush, and carbon rods) materials [21]. This is due to the high conductivity and chemical stability of carbon rods and flake graphite, the huge specific surface areas of carbon cloth and paper, and the corrosion resistance of stainless steel [22]. Flake graphite and carbon rods are also reasonably priced and readily available. However, there are disadvantages to the materials listed as well. For instance, carbon paper is brittle, stainless steel and carbon cloth are expensive, and metallic and carbonaceous materials lack sufficient electrochemical active sites to facilitate the process of extracellular electron transfer (EET) between anode and exoelectrogens [23]. Because of their flat surface, which decreases contact area and makes them less compatible with electrochemically active microbes, MFCs are relatively ineffective. The main purpose of the anode is to encourage the establishment of biofilms and raise the likelihood of EET because the anodic reaction in MFCs is dependent on both the metabolic rate of the bacteria (biocatalysis) and the efficiency of electrode interfacial charge transfer (electrocatalysis) [24]. Decorating with nanoscale materials successfully enables the EET process from microorganisms to the anode surface [25]. A large concentration of micropores characterizes the optimal nanostructure material. This material’s nanoporous structure can enhance the electronic medium’s direct electrochemistry, resulting in more effective electrocatalysis and biocatalysis. Furthermore, this material’s microporous nature promotes the development of biofilm [26]. In addition, the types of nanomaterials may affect the electrode’s homogeneity, surface hydrophobicity, and surface charge, all of which may influence the interaction between the anode and bacteria and ultimately the MFC's capacity to produce electricity[27]. The present review offers a thorough understanding of the applications of nanotechnology and how improving the anode modification of MFCs can improve their performance and efficiency in the production of electricity and the treatment of wastewater.

## Bioelectrochemical systems (BESs) as green technology

Electrochemically active bacteria (EAB) play a crucial role in BESs, which are versatile technologies for energy generation [28]. In BESs, the anodic and cathodic compartments are separated by a proton exchange membrane (PEM). Electroactive microorganisms at the anode oxidize electron donors and reduce electron acceptors. Electrons are transferred from microbial cells to electrodes through electron shuttles in the bulk solution or extracellular components. To maintain electroneutrality, ions pass through the PEM between compartments [29]. The BESs are primarily employed to treat wastewater and produce bioenergy. BESs exceed standard biologic treatment strategies by degrading different types of organic pollutants, enabling treatment and resource recovery [30]. Bacteria can electrochemically convert inorganic carbon to biofuels and value-added chemicals, including biohydrogen, biomethane, acetic acid, and alcohols, using external energy [31]. This feature holds a chance to offer a substitute for using fossil fuels to generate energy and hazardous substances in producing chemical products. Unfortunately, the efficacies of treatment and recovery are insufficient to provide high-quality effluents for possible reuse and energy self-sufficiency. BESs have been applied in many industries and for various purposes: (1) microbial electrolysis cells (MECs) produce hydrogen gas at the cathode [32], (2) microbial fuel cells (MFCs) break down organic material and produce electricity [33], (3) Microbial Electrosynthesis Systems (MESs) that synthesize commodities and value-added chemicals at the biocathode [34], (4) Microbial Desalination Cells (MDCs) that enable water desalination from brackish or seawater, and (5) Microbial Solar Cells (MSCs) that use solar energy to generate electricity [35].

### A brief history of BESs

Luigi Galvani is regarded as the father of bio-electrochemistry. He found that an electrical spark could cause the muscles of a dead frog to contract, and he named this phenomenon “animal electricity,” the force that caused the animal’s muscles to contract [36, 37]. However, Potter discovered 1911 that adding a platinum electrode to a liquid suspension of yeast and *Escherichia coli* allowed the bacteria to produce electricity [13, 38]. Cohen verified this fact later in 1931 when he generated a voltage of 35 V at 0.2 mA of current using a stacked bacterial fuel cell system [13, 39]. Although early studies laid the groundwork for MFCs, it was not until 1963 that the technology gained significant attention. NASA proposed that MFCs could be used to convert human waste into energy during space missions, sparking initial interest [40]. Despite this intriguing application, the technology did not gain much traction, as subsequent proposals, such as using MFCs to power biosensors or self-sustaining robots, lacked clear, targeted objectives [41]. It was not until later that interest in MFCs surged, leading to a wave of research that uncovered affordable materials and clarified the core bio-electrochemical mechanisms behind the technology [42]. Recently, most of the studies focused on investigating the anode side and gave the cathode side sole consideration concerning the oxygen reduction reaction [43]. A number of additional combinations, potential reactions, and high-value products emerged as research into MFCs continued, extending the concept of MFCs to MESs or BESs [44].

### Principles of BESs

The oxidation of organic molecules in the anode compartment produces protons, electrons, and CO_2_ when microorganisms in the BESs promote electrochemical processes. They often build biofilms on the electrode surface to aid in the transmission of electrons [28]. In a PEM or directly, protons pass through the cathode compartment, while electrons go via the anode compartment, where the surface of the anode serves as a terminal electron acceptor (TEA) for the microbes. When these electrons reach the cathode surface, they are reduced to water by oxygen, which acts as the TEA, owing to an external circuit. Electrons flow via the external wire, eventually producing electricity [45]. Several factors, including pH, temperature, reactor design, etc., affect the voltage [46]. To transfer electrons from a solution to an electrode, electroactive microbes (EAMs) are used. The metabolic pathways of these microbes include a number of oxidation and reduction reactions that are associated with internal and EET [47]. They can transfer electrons either directly to extracellular insoluble electron acceptors or indirectly through the use of mediators. The electrical current generated at the anode surface leads to the formation of electroactive biofilm (EABF), composed of colonies of these EAB [48].

### Classification of BESs based on the catalyst used

An electrochemical system consists of many electrochemical devices or copies that rely on different catalysts. A collection of biotechnology-related technologies that are used to produce valuable goods and electricity are referred to as “BESs” technologies. They fall into two groups according to the catalyst that is used. Enzymes are used as catalysts in the first, whereas microorganisms are utilized in the second [49].

### MESs

**Fig. 1:**
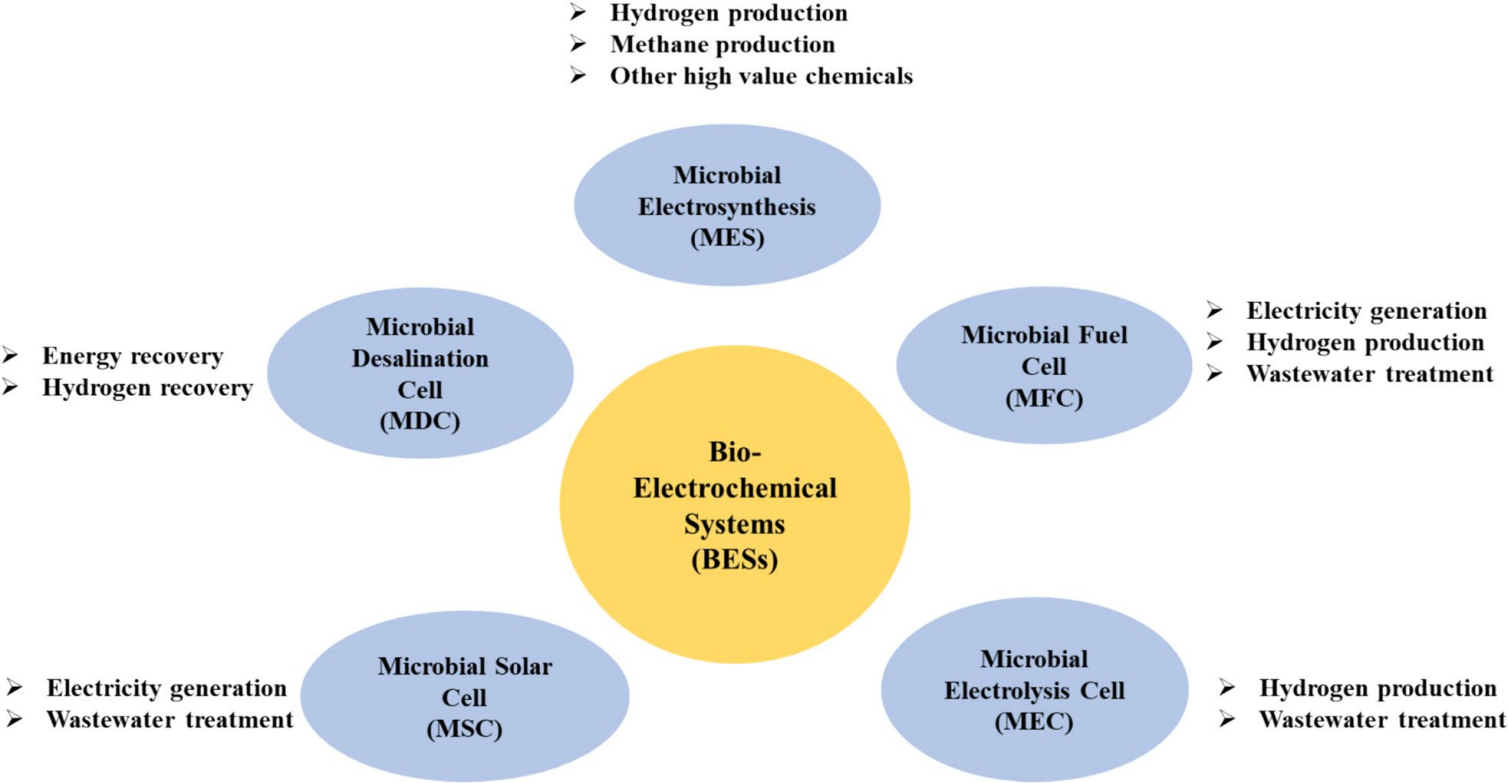
An overview of various types of BESs

There are three main categories of MESs: MFCs, MECs, and MESs. A few other forms, including MSCs [50], plant microbial fuel cells (PMFCs) [51], and MDCs [52], Substrate oxidation in the anode chamber leads to anode–cathode electron transport in MFCs [53] (Fig. 1). Because of their rapid rate of degradation, many MFCs may create bioelectricity by employing a range of carbon-rich wastewater as potential substrates for the goals of pollution reduction and energy generation [54].

### Enzymatic electrochemical systems (EESs)

Enzyme electrodes function as external electron donors or acceptors. EESs consist solely of the enzymatic reaction with a predetermined electron transfer kinetic potential and are frequently utilized in in vitro studies of electron transfer processes [55]. It would be an affordable and accessible strategy to evaluate enzyme reaction kinetics as electrochemical techniques have been utilized to study biologic electron and ion transfer with the most excellent care and precision [56]. When NADH was immobilized on the electrode as the only electron acceptor, electron and proton transfer occurred in vivo [57]. Moreover, the procedures for electron transmission and overall production are still in their infancy.

## An overview of MFCs

**Fig. 2:**
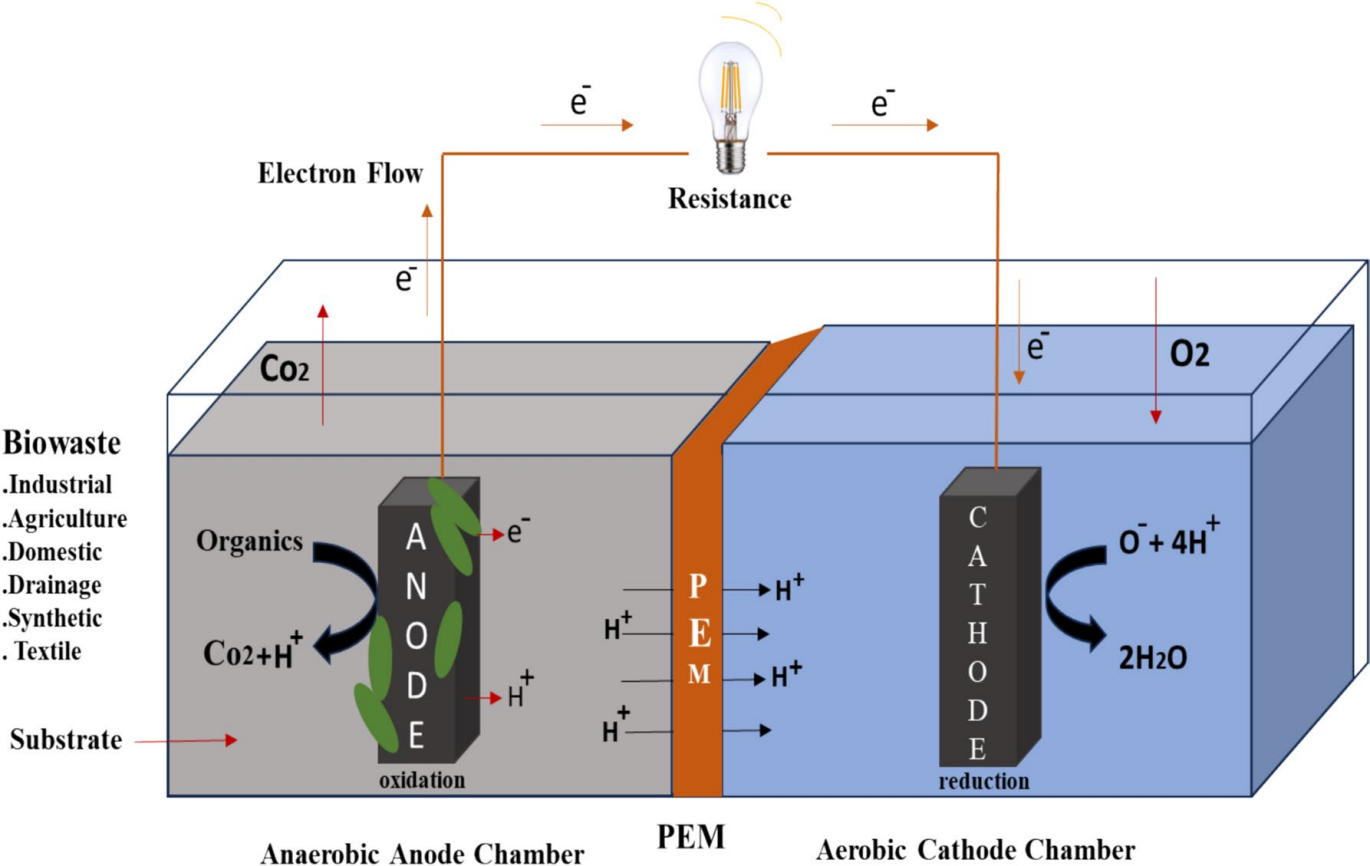
Dual chamber MFC

As illustrated in Fig. 2, the MFCs are innovative and versatile technology that is cost-effective, sustainable, and environmentally friendly [58]. Classified as a form of sustainable energy production, the MFCs uniquely integrate bioelectricity generation with wastewater treatment through the microbial oxidation of organic compounds [59]. The MFCs typically consist of one or two chambers separated by a membrane. The anode and cathode electrodes are housed in separate compartments filled with an aqueous solution, with the membrane serving to prevent the diffusion of electrolytes between the chambers [60]. The anode chamber is designated for the positive electrode, whereas the cathode chamber is allocated for the negative electrode. The solutions in these chambers are called catholyte and anolyte, respectively. The cathode and anode electrodes are interconnected by an external circuit, enabling the flow of electrons and consequently producing an electric current [61]. Protons migrate through the PEM to the cathode, while electrons travel from the anode to the cathode via the external circuit. The difference in redox potential between the anodic and cathodic chambers drives the electron flow. In the anode chamber, bacteria oxidize chemical or organic waste, producing protons and electrons, which play a crucial role in the analysis of organic compounds present [62]. The reaction occurring in the anodic chamber is represented by Eq. (1).

1 $${\text{n CH}}_{{2}} {\text{O }} + {\text{ n H}}_{{2}} {\text{O }} \to {\text{ n CO}}_{{2}} + {\text{ 4n e}}^{ - } + {\text{ 4n H}}^{ + } \left( {\text{Anodic reaction}} \right)$$

Oxidative conditions are created when oxygen is given to the cathode chamber. Electrons and protons are consumed in the cathode compartment. Electricity is eventually produced as a result of the constant passage of electrons. Oxygen is frequently referred to as the electron acceptor in cathodic reactions in MFCs, as in Eqs. (2 and 3) [63].

2 $${\text{n O}}_{{2}} + {\text{ 4n e}}^{ - } + {\text{ 4n H}}^{ + } \to {\text{ 2n H}}_{{2}} {\text{O}}\left( {\text{cathodic reaction}} \right)$$

3 $${\text{n O}}_{{2}} + {\text{ 2n H}}^{ + } \to {\text{ n H}}_{{2}} {\text{O}}_{{2}}$$

The overall reaction in MFC is as in Eq. (4) [64].

4 $${\text{n CH}}_{{2}} {\text{O }} + {\text{ n O}}_{{2}} \to {\text{ n CO}}_{{2}} + {\text{ n H}}_{{2}} {\text{O }} + {\text{ biomass }} + {\text{ bioelectricity}}\,({\text{Overall reaction}})$$

The anode and cathode are particularly critical, as their material type, electrical conductivity, biocompatibility, surface area, and non-toxicity to bacteria significantly affect the MFC’s overall performance [65]. These elements play a vital role in electron transfer as well as in the efficiency of oxidation and reduction processes of organic matter, ultimately influencing the generation of electric power [66]. The PEM is crucial for the optimal operation of the MFC. The choice of membrane material and pore size is critical, as these factors directly influence proton transfer and reduce oxygen transport to the cathode [67].

### Components of MFCs

The main elements of MFCs required for their functioning are the anode, the cathode, the microbial population, and the PEM that acts as a barrier between the compartments. This section comprehensively discusses the many types of components used in MFCs [68].

### Anode

In MFCs systems, the performance of the anode plays a pivotal role in determining the maximum power output that can be achieved. Various materials can be utilized for the anode, including conductive polymers, carbon-based materials, and nanocomposites [69]. Incorporating nanomaterials into the anode design aims to enhance electron transport between the anode and the microbes that serve as biocatalysts in the anode chamber, ultimately boosting power output. These nanostructured materials can either serve as the primary anode material or modify the surface of anodes made from various other materials [70]. Different nanomaterials that used as an anode have been mentioned in Table 1.

**Table 1:** Nanomaterials used as anode materials

Nanomaterials	Power output	Refs.
CNTs + carbon paper	290 mW/m^2^	[71]
Graphene nanomaterials	2668 mW/m^2^	[72]
Carbon nanofibers (CNFs)	36.2 W/m^2^	[73]
Fe_3_O_4_ NPs	728 mW/m^2^	[74]
TiO_2_ NPs	2.6 mW/m^2^	[75]
Polyaniline and CNTs	42 mW/m^2^	[76]

### Properties of anode materials

Some of the following qualities of the anode are necessary to achieve the desired outcomes in terms of electron conversion, bacterial adhesion, and electrochemical efficacy [77].

### Electrical conductivity

The anode material facilitates the flow and acceleration of electrons by receiving and transmitting electrons released by bacteria to the cathode via the external circuit [78]. Lowering the majority of solution resistance and enhancing electron transport are made possible by highly conductive materials [79].

### Surface area

According to Sakai et al*.* [80], anode surface area significantly affects MFCs power output. Using a liquid pulse injection method, Sakai created CNFs of various sizes and examined the nanofibers’ length and diameter effects on the material’s surface area, permeability, and electrical conductivity [81]. The anode’s surface area greatly influences the MFC’s performance since biologic reactions happen there [82].

### Biocompatibility

The anode is in intimate contact with bacteria and their respiration process, making it biocompatible and essential to MFC operations. Due to their susceptibility to corrosion, some materials, such as copper, silver, and gold are not considered biocompatible materials to be used as anode in MFCs [83]. These compounds’ toxicity may inhibit microbial activity during MFCs operation, reducing the amount of energy produced [84].

### Stability and durability

The prolonged exposure of traditional anode electrodes to the substrate and the introduction of microorganisms in MFCs usually result in swelling owing to their lack of compatibility with living organisms, chemical instability, and mechanical instability. The physical stability of the anode is, therefore, weakened. Swelling occurs due to thermal instability, corrosion, and inadequate mechanical strength [84].

### Ease of access to materials and cost

The expense and accessibility of anode materials are crucial since they directly impact the total cost of MFCs. Platinum, gold, and silver are very precious and elusive. Metallic and natural carbon compounds may be attractive alternatives to expensive metals as anode materials in MFCs [85].

### Cathode

In MFCs, the cathode may be built of several materials, much like the anode. Here are some regularly used cathode materials: carbon-based materials, conductive polymers, nitrogen-doped graphene, platinum (Pt) materials, and Pt-based compounds [86]. The cathode facilitates the production of the end product, such as H_2_O, via the combination of protons and a final electron acceptor, such as oxygen. The materials used for the cathode significantly influence the kinetics of the oxygen reduction reactions (ORR) occurring in the cathode chamber. The main factors affecting the ORR include the surface area, electrical conductivity, chemical stability, and activity of the cathode materials, as well as additional pertinent characteristics. Therefore, it is crucial to carefully choose and modify the cathode materials to optimize and improve the performance of MFCs [87]. The advantages and disadvantages of electrode materials are shown in Table 2.

**Table 2:** The advantages and disadvantages of some electrode materials

Electrode materials	Advantages	Disadvantages	Refs.
Stainless steel	High conductivity, low cost, easy accessibility	Poor bacteria attachment, low power production, low surface area, biocompatibility corrosion	[88]
Carbon cloth	High conductivity, flexible, high porosity, high specific surface area	Expensive	[89]
Carbon paper	High conductivity	Low specific surface area, expensive, fragile	[90]
Graphite fiber brush	High conductivity, high porosity, flexible, high specific surface area	Expensive, clogging	[91]
Graphite felt	High conductivity, flexible, high Specific surface area	Low strength	[92]

### Proton exchange membrane (PEM)

The main factor influencing MFC efficiency is PEM. Oxygen crossing from the cathode and substrate transfers from the anode to the cathode are stopped by the membrane. Protons can move selectively from the anode to the cathode thanks to its additional function as a physical conductor or separator [93]. Nafion and Ultrex, two popular commercial MFC PEMs, require high proton conductivity, low internal resistance, good energy recovery, and great chemical and physical stability [94]. Owing to these limitations, there is currently a trend toward using nanomaterials in membrane topologies to enhance membrane performance as well as their mechanical and thermal properties. This enhances the performance of MFCs and helps achieve the required qualities [95]. For instance, it can be applied to decrease proton permeability and the mechanical and thermal stability of the membrane [96].

### Degradation of organic pollutants’ effects on power output

Numerous crucial elements can impact the energy efficiency of MFCs, including the material employed as the anode, the microorganisms’ electroactivity, external resistance, and the organic substrate that the microorganisms depend on for development and respiration [53]. It makes sense that the breakdown of organic pollutants by microbes will enhance the energy efficiency of the MFCs since bacteria may also use them as a source of carbon [97]. But this depends on the organic pollutant’s concentration, not damaging the microbial ecosystem [98]. Numerous investigations demonstrate that microbial communities capable of consuming hydrocarbon pollutants as a substrate can also move electrons to the MFC's anode electrodes to produce power [99]. This is significant because it suggests that in-situ bioremediation of organic pollutants in groundwater can be enhanced by applying the MFC approach [100]. Research has indicated that the gradual deterioration of organic pollutants leads to a higher energy output in MFCs [101].

## Factors affecting the performance of MFCs

### Temperature

Numerous studies have previously demonstrated that temperature is a factor that can significantly affect MFCs efficacy in a variety of scenarios. According to Adelaja [102], the impact of temperature on the decomposition of various hydrocarbon compounds in petroleum wastewater and power production using MFCs dual chamber in semi-batch mode has been investigated. Conversely, performance decreased at 50^◦^C, indicating that 40^◦^C is the ideal temperature for MFCs operation when supplied petroleum hydrocarbon-contaminated effluent. The quantity of organisms that can survive in the anodes decreases at very high or low temperatures, subsequently affecting the MFC’s overall efficiency. The majority of bacteria prefer to adapt to mild temperatures, which explains this tendency [103].

### External resistance

The external resistance significantly affects the removal capacity of various elements in MFCs [104, 105]. External resistance may directly impact the anode potential, which influences the development of the electrochemically active biofilm and MFCs performance. Selective electricigens are encouraged by low external resistance since they boost the electron flux and allow the EABF to have greater free energy flow [106]. Low external resistance results in higher power outputs and lower chemical oxygen demand (COD) removal efficiency, whereas high external resistance is linked to higher COD removal and lower power output [107].

### pH

Based on studies, pH significantly affects the ability of microorganisms to produce products, metabolize substrate, develop as a community, and also affect their redox potential [108]. In a study carried out by Sogani et al. [109], EET performed well at acidophilic pH, and they hypothesized that this could be because of specific intracellular electron transfers that help rearrange electrons. In neutral and alkaline pH settings, hydrogen ions (H^+^) tend to decrease during substrate degradation, releasing fewer protons and electrons [110].

### Substrate

One of the primary causes of the formation of various microbial communities in BESs is the diversity of substrates used. A microbial community that has been injected in BESs develops a tolerance to a particular substrate, and altering the substrate has an impact on the microbial population [111]. According to Sonawane et al. [112], glucose, butyrate, starch, cellulose, and phenols are just some of the carbon-rich organic substrates that can be utilized for microbial metabolism. It is common to use lignocellulosic biomass as a substrate to increase the output of MFCs. Carbohydrates are abundant in carbon-rich wastewater from starch processing, which qualifies it for MFCs technology [113, 114].

### Electron transfer mechanisms in MFCs

MFC is a bio-electrochemical system made up of an anaerobic anode chamber and an aerobic cathode chamber separated by a PEM or salt bridge. Microorganisms in an MFC oxidize substrate-producing electrons, which are then taken by the anode electrode and moved to the cathode via an external wire. Protons diffuse across the PEM. Water is produced when electrons, protons, and oxygen combine at the cathode [42]. Microbes that can exchange electrons with BES electrodes have specific biologic characteristics or produce metabolites in response to environmental factors and their genetic makeup [115]. *Geobacter* and *Shewanella* sp., were thoroughly investigated for their EET. EET can occur through two main routes: direct and indirect [116].

### Direct electron transfer (DET)

**Fig. 3:**
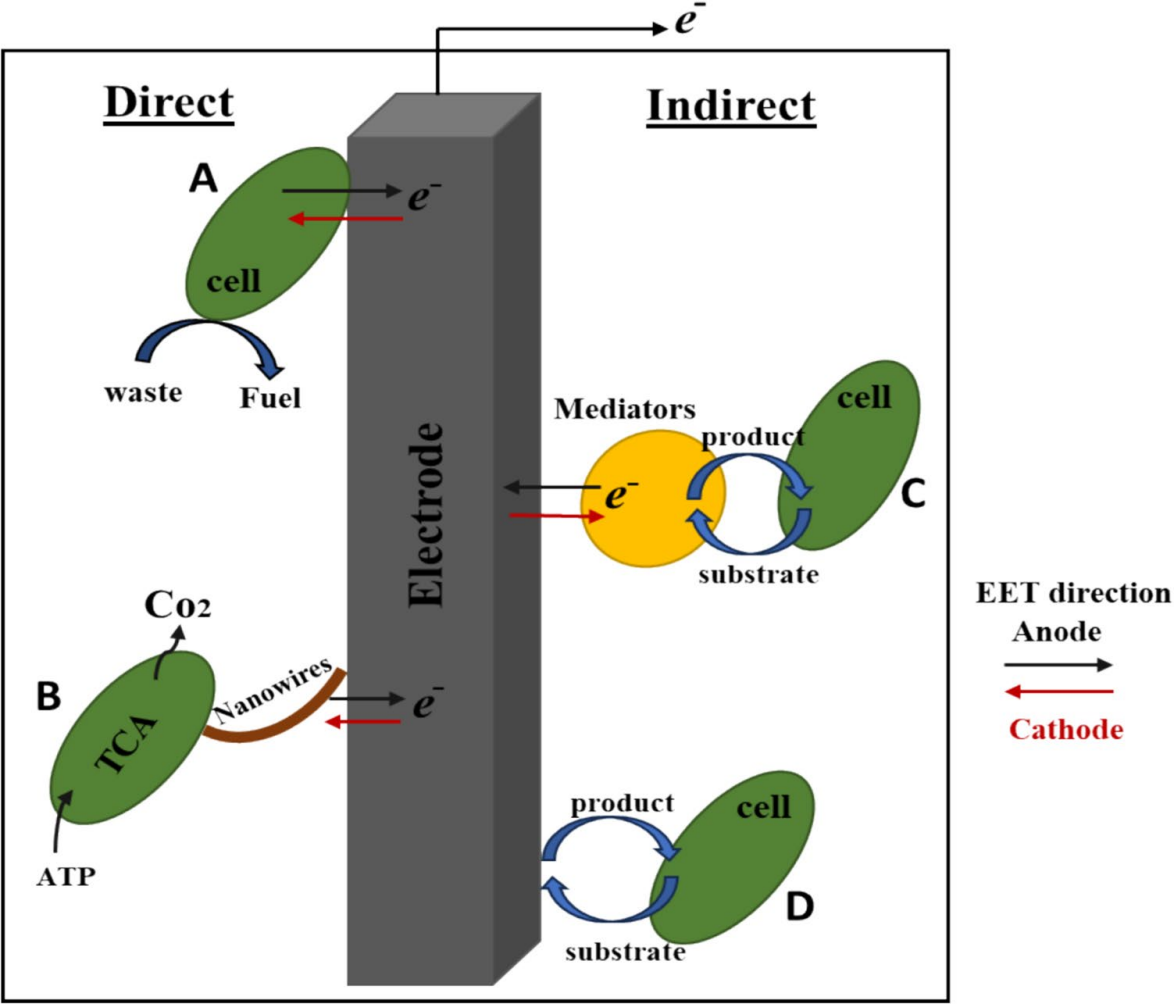
Different EET processes A-Direct EET B-Nanowires direct EET C-Mediated EET D-No EET

Microbes can transfer electrons directly to the electrode surface through DET in the absence of a mediator (Fig. 3). DET to the electrode is possible for bacterial species that respire next to the electrode surface and have exposed c-type cytochromes in their outer cell membrane [117]. *Enterococcus gallinarum, Shewanella putrefaciens, Geobacter sulfurreducens, and Geobacter metallireducens* were discovered to possess this mechanism [17]. Approximately 111 genes in *Geobacter* and 42 genes in *Shewanella* are related to c-type cytochromes [118].

### Indirect electron transfer (IET)

The EAB in the BESs eventually spread away from the electrode surface as biofilm forms at the anode surface, making it more difficult for them to transmit electrons. The indirect electron transfer is accomplished by creating a mediator or shuttle (Fig. 3). Redox mediators are supplied externally or secreted by microbes. To overcome this restriction, artificial mediators such as thionine, phenothiazines, neutral red, phenoxazines, benzyl viologen, etc., were added to the system [119]. Some bacteria can secrete mediators like quinone and flavin, which function as electron shuttles [120]. Mediators work well when the electroactive microbe is at the outermost layer of the EAB or farthest from the electrode in a planktonic culture. All these element — nanowires, cytochromes, nanowires, and mediators—maintain the conductive nature of EAB [17].

### Formation of EABF

A microbial aggregation membrane, or biofilm, is created when microbes adhere to the surfaces of biotic or abiotic objects. As “protective clothing,” biofilm allows bacteria to tolerate harsh conditions and can shield them from ultraviolet radiation, severe pH, extreme temperature, high salinity, high pressure, starvation, antibiotics, and other threats [121]. Biofilm is microbial growth’s self-defense mechanism [122], and it also seems that the three-dimensional structure of biofilm offers bacteria a built-in barrier and protective layer [123].

### Multiple phases involved in the production of biofilm

**Fig. 4:**
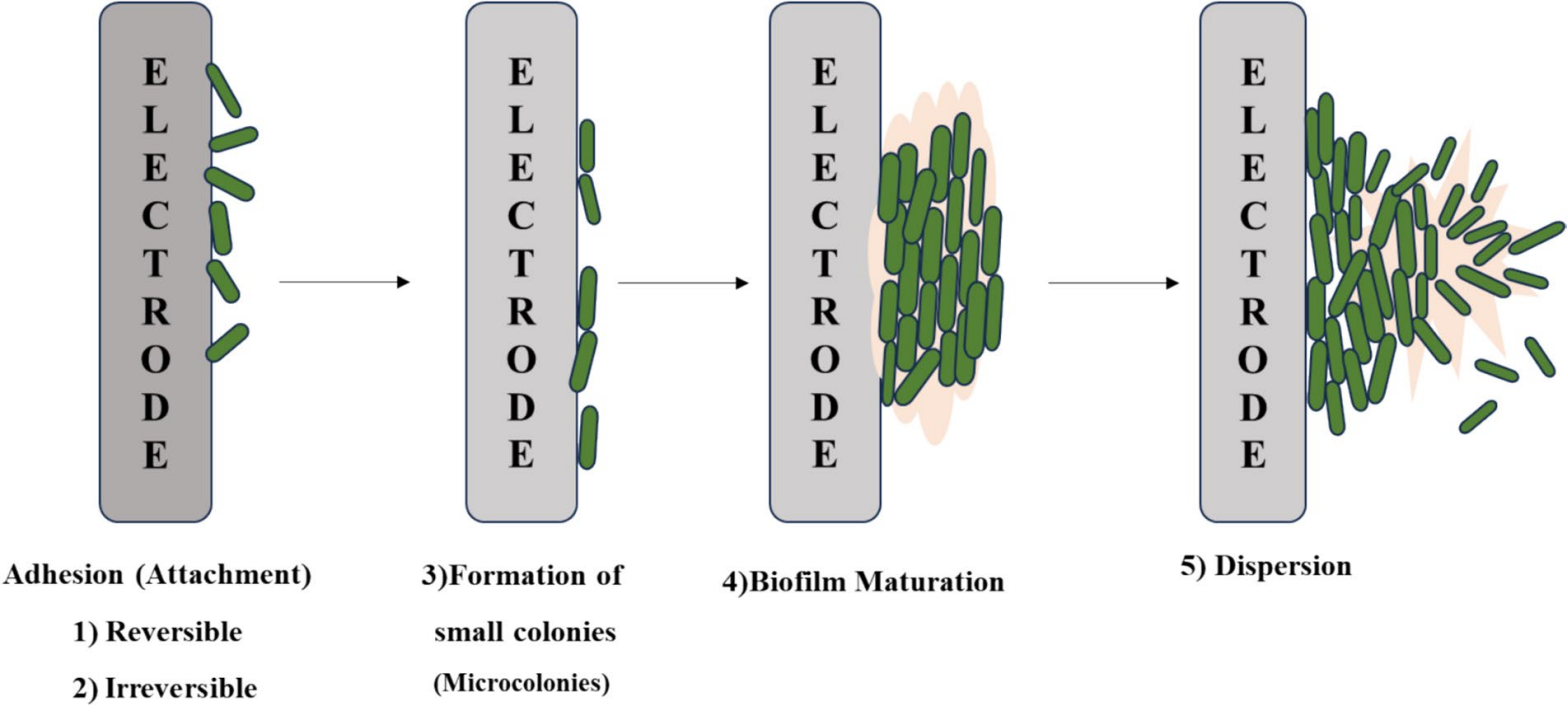
The five fundamental steps that result in the creation and growth of biofilm

**Fig. 5:**
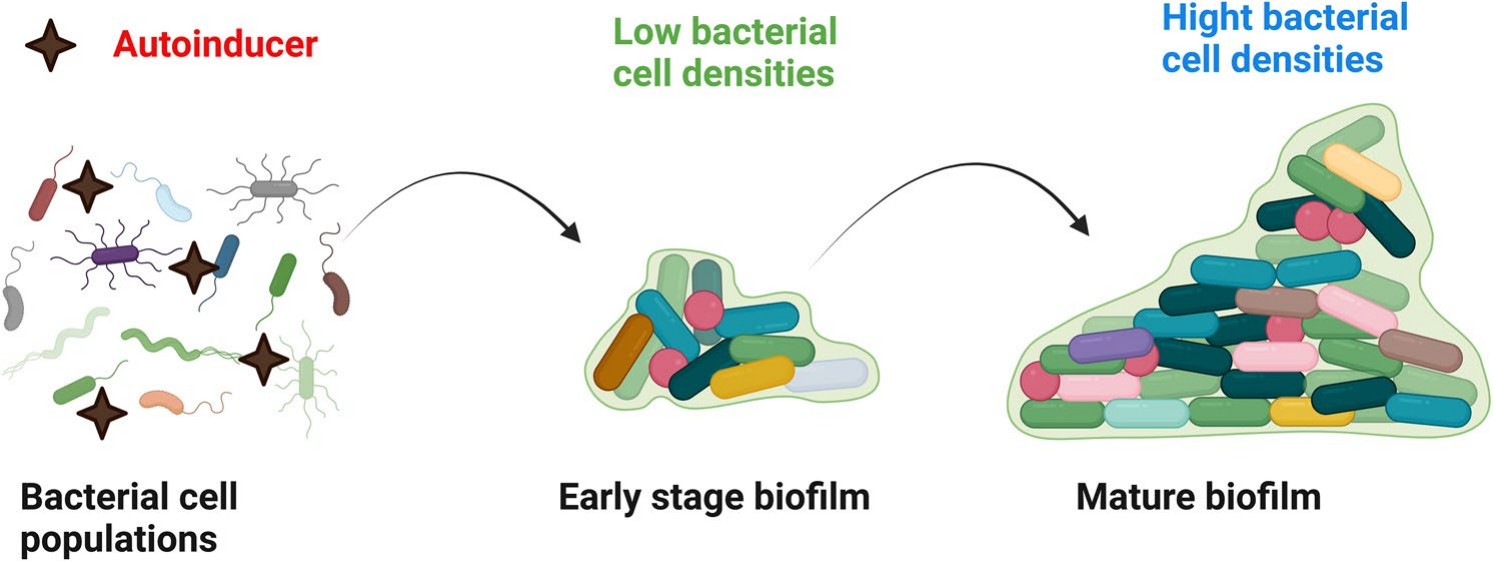
Illustration of QS. A signaling molecule is an autoinducer. The amount of autoinducers rises with the number of bacterial cells since these cells continuously manufacture them

The following phases are involved in the multi-step process of creating a three-dimensional biofilm structure: (1) reversible attachment, (2) irreversible adhesion, (3) initial biofilm structure development through the generation of tiny colonies, (4) biofilm maturation, and (5) dispersion as shown in Fig. 4 [124]. Using organic molecules (such proteins, lipids, polysaccharides, and fatty acids) or inorganic substances (like water and inorganic salts), bacteria start the process of producing a biofilm by forming a suitable surface layer [125]. A mixed structure of extracellular polymers, either in single or combination populations, then embeds this biofilm [126]. Once bacteria have attached to a biotic or abiotic substance, they communicate with one another through an extracellular signaling mechanism based on quorum sensing (QS) [127]. QS can control each step of the biofilm growth process by causing certain genes in bacteria to create extracellular matrix-like proteins and extracellular polymeric substances (EPSs) and progressively building a fully formed biofilm structure[128]. he maturity of the biofilm is also controlled by QS intercellular communication. The production, release, and accumulation of extracellular signal molecules are mediated by the chemical interaction between bacterial cells, or QS Self-inducers are molecules that act as chemical signals [129]. These self-inducers are continuously produced by bacterial cells, thus their quantity rises as the number of cells does (Fig. 5). This process may have a role in controlling the formation of biofilms, particularly in relation to the synthesis of extracellular polysaccharides and the formation of channels or columnar structures [130]. These structures’ development guarantees that nutrients reach the cells in a biofilm community [123]. In addition, to facilitate communication among microorganisms, bacteria typically incorporate the information present in some QS automated induction factors into the regulation of gene expression [131].

### Purpose of EABF in BESs

Biofilm formation plays an important role in the physiology and function of microbial communities, including microbial defense against stressful conditions, host immune system response to invasion, nutrition recycling, organic matter biodegradation, and electron transfers in BES [132]. EAB are microbial biofilm having the ability to exchange electrons with external electrodes. EAB oxidizes substrates and stores electrons in the form of reduced flavins, cytochromes, quinones, and polyhydroxyalkanoates [133]. When the external circuit is linked again, the stored charge is released. It is also known that BES operation, that is, this sporadic alters the morphology of EAB [134].

### Parameters influencing the production of EABF

**Fig. 6:**
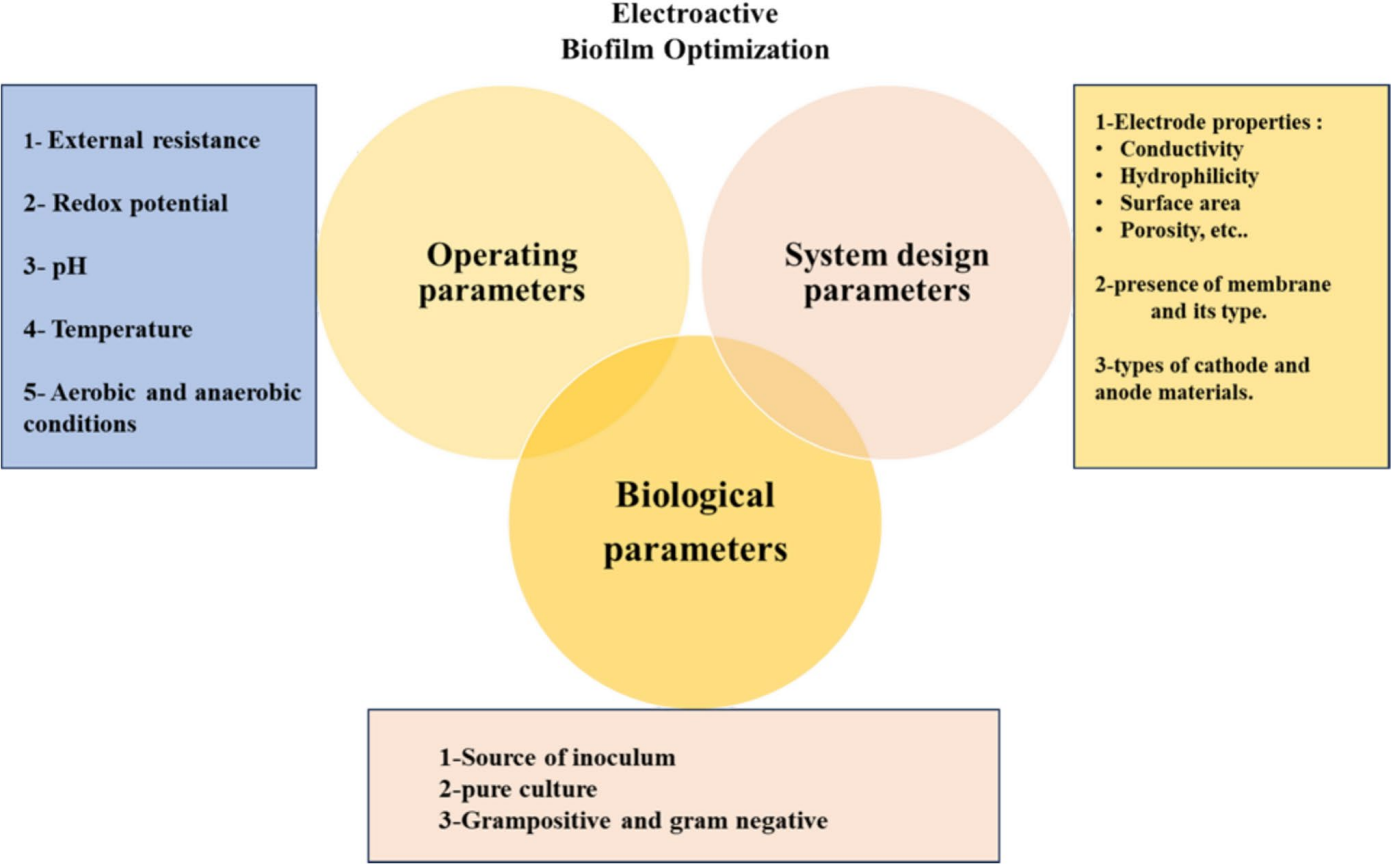
Factors influencing the production of EABF in BESs

The production of biofilm in BESs can be influenced by a wide range of variables. Generally, biofilm formation can be influenced by three primary criteria: biologic characteristics, system construction, and operating variables. Figure 6 shows these variables that are mentioned.

### System construction

Since the electrode surface is where electron exchange takes place, the electrode material is essential to the effective operation of EABF. Because different electrodes have different conductivity, surface area, and porosity, the relationship between the microbe and the electrode is shaped by this variety. Effective microbial attachment requires the electrode to have certain qualities, such as high conductivity, chemical stability, a large surface area, biocompatibility, high porosity, and cost [135]. In the BESs, carbon cloth, carbon felt, carbon paper, carbon mesh, carbon brush, graphite rod, graphite felt, etc. are the electrodes that are most often utilized [136]. It was discovered that in some experiments, utilizing carbon nanotubes (CNTs) as an electrode increased power output; nevertheless, it also causes bacterial cell death [137].

### Biologic parameters

Gram-positive or Gram-negative bacteria, the type of culture (pure or mixed), and the inoculum’s source are all important for the development of EABF. Gram-positive bacteria have been shown to produce distinct factors that impact the EABF within the BESs [138]. According to Borole et al. [139], the inoculum source is essential to the system's microbial community development.

### Operating parameters

The formation of EABF in the BESs is influenced by several operational parameters, including pH, temperature, shear rate, presence of oxygen, substrate concentration, external and internal resistance, and so forth [140].

### Scaling-up applications of MFCs

From lab-scale to commercial applications, MFCs demonstrated their efficacy as a treatment technique for pollutants eliminated from industrial discharges, includes those from the food processing, dairy, paper and pulp, leather, brewing, distillery, and textile industries, etc. MFCs are efficient tool for treating the pollutants that found in industrial outputs, such as those from textile, brewing, distillery, leather, pulp and paper, dairy, and food processing sectors. This applies to both lab-scale and commercial applications [80]. Following a few fruitful efforts in field applications over the last 20 years, several industrial firms and young businesses suggested applying MFCs for industrial wastewater treatment on-site (Table 3) [141].

**Table 3:** Applications of MFC technology in industry

Industrial applications	MFC configuration	Reactor volume (*L*)	Microorganism	Substrate	Anode electrode	Power generation	Refs.
Bioelectricity production	Sedimental MFC	8.00	Mixed culture	Sediment	Copper	0.78 mW/m^2^	[142]
Bioelectricity production	MFC stack	20.00	Mixed culture	Acetate + buffer	Titanium	144.00 W/m^3^	[143]
Wastewater treatment	Dual chamber	1.00	*E.coli* INVaF (Invitrogen)	Synthetic wastewater + potato extract	Titanium	502.00 mW/m^2^	[144]
Wastewater treatment	Single MFC	1.87	Anaerobic mixed culture	Synthetic wastewater	Carbon felt	NA	[145]
Wastewater treatment	Up-flow MFC	4.90	Mixed culture	Tapwater solution	Titanium wire inserting granular carbon	15.46 W/m^3^	[146]

With the use of cutting-edge redox catalysts and materials, Indian Oil Corporation Ltd. (India), Tata Consultancy Services (India), and other are developing and improving the performance of MFCs [147]. Benthic, sedimentary, solid-phase, and up-flow tubular systems are examples of newly modified MFCs utilized in industrial applications [148, 149]. As previously indicated, they are easily incorporated into other contexts [147]. The anode and cathode sections are rearranged throughout construction to fulfill their intended purposes. This situation further exemplifies the importance of setup in expanding the scope of applications for MFCs. Desalination, wastewater treatment, and bioremediation are currently beneficial for MFCs [150]. Various industrial activities in the pharmaceutical, cosmetic, textile, and paper sectors generate waste products include pesticides, organic chemicals, phenolic compounds, nitrogen-based compounds, antibiotics, and artificial coloring, and pollutants. Microorganisms in the MFCs are seen as sources of nutrients such as carbon, nitrogen, phosphate, etc., which they may use to generate energy [151]. Because they do not require substrate and substrate-feed energy, these areas can lower the cost of energy generation [152].

## Electrochemical characterization techniques

### Cyclic voltammetry (CV)

CV is the most widely used technique for gathering qualitative and quantitative information about electrochemical reactions [153]. The CV method is rapidly becoming a standard methodology in the study of MFC and seems to be receiving a lot of attention. Identifying the redox potential of the species involved under different operating conditions, characterizing the mechanism of electron transfer between the biocatalyst and the bioanode or biocathode, and interpreting the anodic electron transfer at different stages of microbial development and metabolic processes [154], are the three main uses of the CV method in MFC studies. Bhuvanendran et al. [155] were the first to report on the feasibility of using CV as a transduction method for biofilm identification.

### Electrochemical impedance spectroscopy (EIS)

EIS is an efficient and non-destructive way to study the bio-electrochemical processes of MFC [156]. With electrochemical impedance spectroscopy, or EIS, different electrochemical processes can be examined at different frequencies. By placing a small, variable-frequency sinusoidal alternating (AC) potential on top of a direct current (DC) potential, the amount of current flowing through the cell may be determined. A pseudolinear trend of the impedance is obtained for the generally non-linear electrochemical systems by adding a small AC potential, usually between 1 and 10 Mv [157]. Essentially, this technique uses two electrodes to identify variations in the solution resistance caused by byproducts of microbial metabolism [158]. However, EIS can change as a result of microbial metabolism and microbial cells adhering to the working electron (WE) [159].

## Recent advancements in nanotechnology applied in the modification of MFCs anodes

### Carbon-based nanomaterials

Decorating the interface between anodes and exoelectrogens is necessary, carbon-based nanomaterials are commonly utilized. They not only possess the advantages of conventional carbon materials but also display a high surface area, which are beneficial for bacteria attachment and increase the number of contacting active sites for EET [160].

### Carbon nanofibers (CNFs) and Carbon Nanospheres (CNs)

Organic waste and glucose are two examples of natural and recyclable materials that are frequently used in the manufacturing of high-performance CNFs and CNs [73]. By carbonizing and activating organic matter at high temperatures, a solid phase is created that produces nano-spheres with consistent diameters, improved appearance, and a larger surface area. In addition, in long-term operation, the produced nanosphere shows good electrical conductivity and biocompatibility [161]. CNs improved bacterial adhesion active sites resulted in higher electron transfer rates and a notable boost in energy density, indicating that their inexpensive cost may make them useful in real-world applications [162].

Nanofiber technology, which has a number of benefits over conventional electrode materials, is being used to advance the development of MFCs. Composites, polymers, metals, and ceramics are some of the materials that can be used to create nanofibers, which are extremely thin fibers with diameters between 1 and 100 nm [163]. Nanofibers can be used as electrode materials in MFCs because of their porosity, small diameter, and high surface area-to-volume ratio. Because there are more microbes adhering to the electrode, performance improves [164]. Nanofiber’s small diameter makes it easier for microorganisms to reach the electrode surface, which enhances their metabolic activity and increases energy production. The growth and metabolism of microorganisms depend on the transport of nutrients and oxygen, which is made easier by the high porosity of nanofiber[165]. Furthermore, some nanofiber materials—such as graphene (Gr) and CNTs—have high electrical conductivity, which makes it easier for electrons to move between the microbes and the electrode and improves energy production [166]. When conductive nanocomposite was added to the anode of MFCs, the power density increased by 1.8 times in comparison to commercial graphite felt. Carbon nanofiber's influence on MFCs stems from both its adoption qualities and conductivity. The conductive carbon nanofiber can be used to improve extracellular electron transport and bacterial adhesion at the same time [167].

### Carbon nanotubes (CNTs)

Researchers and industry professionals have widely employed CNTs in a variety of cutting-edge applications because of their remarkable mechanical, thermal, and electrical characteristics, whether in their standalone or reinforced composite forms. CNTs’ surface activity can be increased through physical, chemical, or photochemical modification to improve their qualities for certain uses. This surface activity enhancement of surface-modified carbon nanotubes (SM-CNTs) has been used by the researchers to meet a number of their research objectives [168]. Photocatalysts, electrocatalysts, and biocatalysts on CNTs can be stabilized for photochemical and electrochemical applications through surface modification, as evidenced by their dispersion stability in reaction media. SM-CNTs are more compatible with electrolytic membranes and coatings than unmodified CNTs. The performance of electrolyte membranes and electrodes can be improved by using SM-CNTs, which is a promising study field for the development of energy devices. A variety of fuel cells, including microbial, enzymatic, and high-temperature fuel cells, use SM-CNTs to provide clean energy that potentially power the planet. By employing SM-CNTs to catalyze anodic and cathodic electrode processes and enhance conductivity, electrolyte membrane performance can be enhanced [169].

### Graphene nanomaterials

Because of its good catalytic activity, high electron mobility, and acceptable biocompatibility, Gr, the building block of other graphite materials, is a suitable choice for an active anode-enhancing material [170]. This comprises Gr and its compounds, including reduced graphene oxide (rGO), graphene oxide (GO), single/multi-layered graphene, and hybrid nanocomposites [171]. Different graphene-based nanocomposites have been applied in anode material in MFC applications.

The remarkable 2D-carbonaceous electrode nanomaterial such as Gr and CNTs have been used in many different applications because of their improved electrical conductivity, excellent electrochemical stability, and superior biocompatibility. In addition, CNTs made on Gr serve as reliable conducting channels between electrodes and the relevant components [172].

### Metal-based nanomaterials

Although metals are more conductive than carbon, their widespread use is limited by their corrosive properties. Within MFC, nanostructured metal materials are currently employed as anodes due to their superior conductivity, catalytic activity, and biocompatibility. Between the bacteria and the anode, To enhance the anode, nanoparticles act as a link for electron transport. There are active places for the anode’s bacterial growth, which raises the rates at which energy is generated [173].

### Manganese oxide (MnO_2_) nanoparticles

MnO_2_ have garnered a lot of attention because of their excellent catalytic qualities in an electrochemical reaction system, high stability, low cost, and varied oxidation states. Alkaline and rechargeable batteries, sensors, supercapacitors, wastewater treatment, and catalysis have all made use of these fascinating composites [174]. As anode materials for energy conversion and storage device systems, they have also garnered a lot of interest. Since the sizes, phases and characteristics of nanomaterials greatly affect their qualities and uses, a number of innovative and efficient methods have been developed to create MnO_2_ nanoparticles with a variety of shapes and superior qualities [175].

### Iron oxide (Fe_2_O_3_) nanomaterials

By enhancing electrical conductivity and promoting microbial activity, Fe_2_O_3_ raises MFC power production. Because of its ability to enhance the anode’s surface structure and produce electrical energy in the MFC, Fe_2_O_3_ is a material that is appealing for anode electrode modification. According to studies, adding Fe_2_O_3_ to the anode electrode improves the adhesion of microbes by changing the surface from smooth to rough [176]. In addition, because of the hydrogen link that forms between the oxygen atoms of Fe_2_O_3_ and the water molecule, covering the hydrophobic carbon surface with Fe_2_O_3_ can hydrophilize it [177]. his material is a good choice because of its low cost, chemical stability, and accessibility. In MFC configurations, it is commonly used as a modifier due to type C cytochromes (OmcA and MtrC) are strongly attracted to insoluble Fe_2_O [178], which affects the enhanced electron transport. According to studies, the percentage of electroactive bacteria on the anode, including *Pseudomonas* and *Geobacter*, increases when Fe(III) oxide is present [179].

### Titanium oxide (TiO_2_) nanomaterials

In comparison to traditional graphite and spongy materials, TiO_2_ nanoparticles and carbon-assembled core–shell nanoparticles from egg white protein were used to build the anode’s capacitive layer, which produced more electricity [74]. Improved electrochemical activity and synergistic effects with carbon composite nanoparticles produced from egg white protein and TiO_2_ were responsible for the increased power output. These nanomaterials had a high specific surface area and were highly biocompatible. In *Escherichia coli* MFCs, Qiao et al. have demonstrated how to produce a special mesoporous TiO_2_-nanostructured polyaniline (PANI) hybrid and employ it as the anode [180].

### Polymer (polyaniline–pyrrole) nanomaterials

As conjugated conducting polymers, polyaniline and pyrrole both have remarkable properties such as low cost, minimal environmental instability, and great electronic conductivity. Furthermore, it is simple to combine the conductive polymers with other nanomaterials to form nanocomposites [181]. In comparison to traditional carbon materials, the resulting nanocomposite improves electron transmission and lowers Ohmic resistance. Furthermore, the positive charge of polyaniline draws in electrochemically active bacteria, increasing the power generation of MFC [182].

### Anode material and modification

**Fig. 7:**
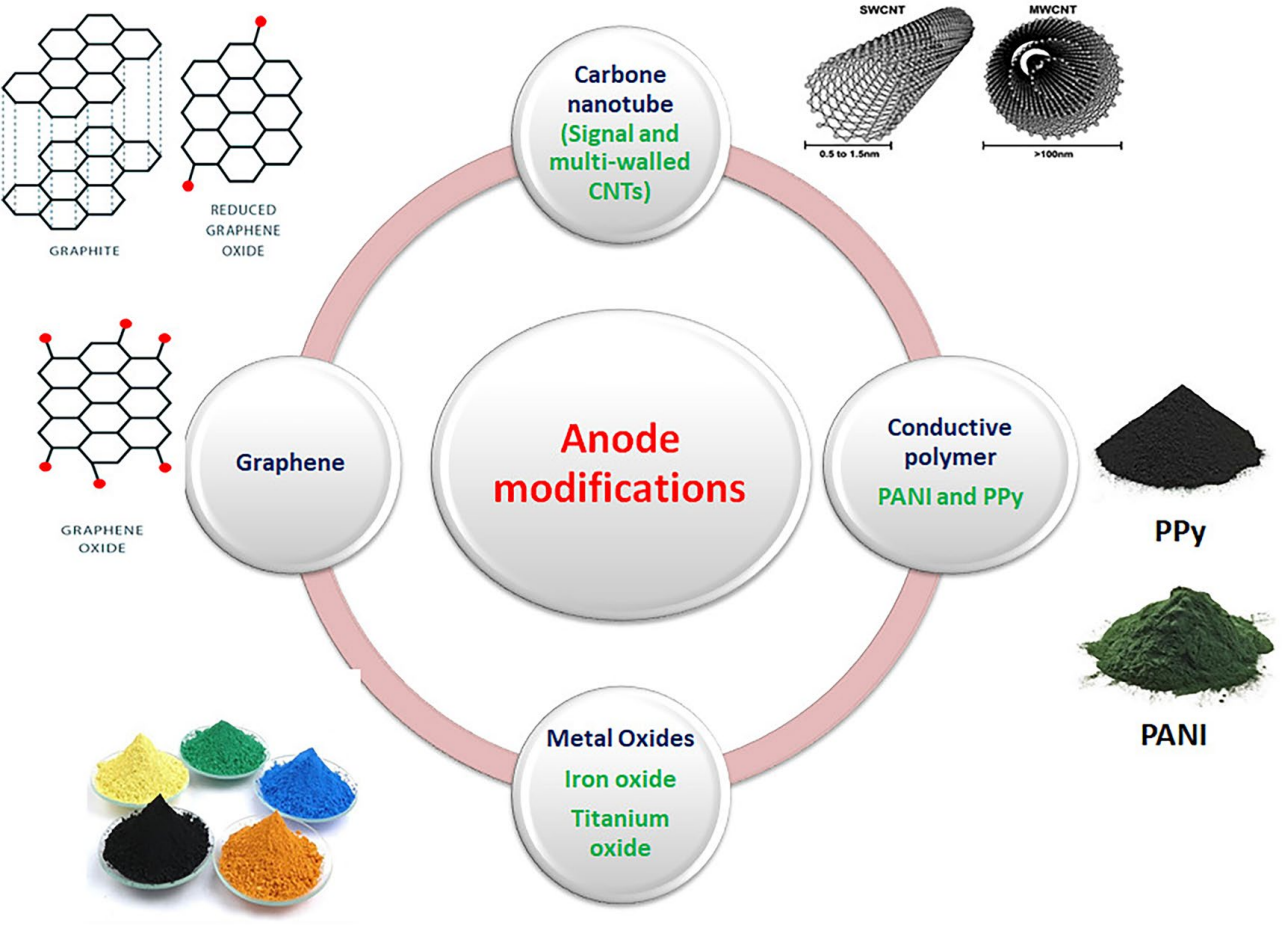
Materials of anode modification to enhance the efficacy of MFCs

The electrodes in MFCs must fulfill specific characteristics to improve the MFC’s performance and achieve high energy recovery. These requirements include: (i) high biocompatibility; (ii) large surface area; (iii) enough electrical conductivity; (vi) strong mechanical strength; and (v) stability and durability [183]. In the scale-up investigation, it is possible to use stainless steel mesh as a current collector. Graphite and carbon electrodes can be used in brush, felt, cloth, rod, granular, reticulated vitreous, and micro-porous forms, as shown in Fig. 7 [65]. According to Banerjee et al. [184], increasing surface area promotes microbial activity and enhances electrode kinetics. A more uneven electrode surface results in a stronger attachment to the biofilm. As a result, increasing the roughness of the electrode’s surface area increases the number of active sites, hence improving the ability of bacteria to attach to the electrode surface. The selection of a conical shaped material based on graphite was made due to its notable benefits in enhancing compactness, reducing electrode interspacing, and offering a vast surface area for the electrodes [185]. Table 4 shows some of the MFC previous scale-up studies.

**Table 4:** An overview of recent MFC scale-up studies

Reactors	Anode	Cathode	Substrate	Membrane	P_max_	CE%	COD removal%	Refs.
Submergible MFC, 255 L	Graphite fiber brush	Stainless steel –activated carbon	Municipal waste water	Glass-fiber separator	0.3	30	41	[186]
Dual-chamber MFC, 1.5 m^3^	Graphite fiber brush	Bio- graphite fiber brush	Primary effluent	Dynamic (microbial) membrane	0.4	–	91	[187]
Dual-chamber MFC, 60 L	Granular graphite	Granular rod	Swine manure	Anionic exchange membrane	4	17	36	[188]
Dual-chamber MFC, 60 L	Stainless steel	Granular rod	Swine manure	Anionic exchange membrane	2	17	40	[188]
Dual-chamber MFC 200 L	Graphite fiber brush	Carbon cloth/N-activated car	Primary effluent	Cationic exchange membrane	1	–	75	[188]
Hybrid anaerobic-anoxic/oxic; single-chamber MFC, 1 m^3^	Graphite fiber brush /Stainless steel	Aerobic effluent carbon fiber brush—stainless steel	Domestic waste water	None	0.0036	–	95	[189]

### Surface modification techniques

The electroactive properties and efficacy of EET in biofilms are controlled by the anode surface's physical and chemical properties [190]. As a result, surface modification methods refer to some of the innovative techniques that can be used to enhance MFCs performance. Physical and chemical treatments comprised most of these categories [191]. Heat treatment, for example, is a physical treatment that is very effective in producing crack surfaces on the working electrode. It accelerates the growth of the electro-active microbial film and intensifies specific active sites, increasing the active sites by almost seven times when compared to non-modified anodes [192]. Similarly, chemical treatments like acid treatment were applied to enhance the functional group characteristics on the working electrode in a way that was advantageous to the growth of microorganisms. This technique results in a large number of electrochemically active sites [193]. The surface modification techniques used for anode (electrode) materials in MFCs research are compiled in Table 5.

**Table 5:** An overview of the surface modification techniques used in the microbial fuel cell studies

Types of MFC	Surface modification	Anode	Cathode	Inoculum source	P_max_ (mW m^−2^)	Refs.
Single chamber	Acid and heat treatment	Graphite felt	Ketjen black	Brewery wastewater combined by domestic wastewater	28.4	[194]
Single chamber	Electrochemical oxidation	Carbon cloth	Carbon cloth	Domestic waste water	937	[195]
Dual chamber	Methylene blue treatment	Graphite felt	Graphite felt	Sulfate reducing bacteria	510	[196]
Dual chamber	Ammonium per oxy di-sulfate treatment	Graphite felt	Graphite felt	Sulfate reducing bacteria	355	[196]
Dual chamber	Electrochemical oxidation	Graphite felt	Graphite felt	Effluent of MFCs	1.13	[197]
Dual chamber	Ethylene di-amine treatment	Graphite felt	Graphite felt	Sulfate reducing bacteria	545	[196]

### Anode modification with conducting polymer

Due to their high conductivity, durability, and environmental friendliness, conducting polymers have attracted a lot of interest as a practical functional anode element. Numerous methods can be used to improve microbial attachment, such as establishing a consistent link with the right pore size [198], boosting the positive charges to produce an electrostatic attraction with exoelectrogens, reducing internal resistance, modifying the electrochemical reaction rate, and raising the functional electrode's specific capacitance [199]. The functional anode feature is frequently enhanced by conducting polymers [200]. The efficiency of the working electrode can also be greatly increased by combining conducting polymer material with nanomaterials as CNF, rGO, Pt NPs, and MWCNT. For the anode surface modification procedure in MFCs, conducting polymers such PANI, poly (3, 4-ethylene-dioxythiophene) (PEDOT), and poly-pyrrole (PPy) were typically used [201].

### Augmentation of the electrode using a material based on metal oxide

The addition of a metal-oxide nanoparticle component significantly increased the total output power by lowering the ohmic resistance and enhancing the microbial community's adhesion [202]. Iron oxides, titanium, manganese, and other elements are also being widely considered as electrode additives. These oxides can be coupled with other working anode electrode materials to make nanocomposites. Tin oxide exhibits remarkable physicochemical properties, including its high abundance, excellent conductivity, relatively low cost, and ease of availability, which render it highly effective for electrode modification [201].

### Anode modification with carbon nanotubes

Because of their exceptional conductivity, high catalytic activity, increased active sites, and superior electrochemical stability, CNTs provide a plethora of opportunities and chances as an anode additive. Furthermore, adding CNTs to particular materials may make it easier for porous structures with lower porosity to form. By strengthening the material's working electrode’s active site, these additions increase the material's electro-catalytic activity, electro-chemical stability, and electron transfer efficiency [203]. For single-walled and multi-walled CNTs, the open ends and closed ends, respectively, are associated with the tubular structure feature of the carbon nanotube. The mechanical strength, conductivity, and structural flaws of both types of CNTs are distinctive. Although some researchers have proposed that by inhibiting microbial growth, CNTs have a detrimental effect on the microbial population [204], The application of CNTs in working anodes in MFCs technology has resulted in countless improvements and numerous advantages that have made it feasible to lower the undesirable biotoxicity by modifying and controlling the quantity of CNTs that bind with particular functional group elements or otherwise assimilate with specific conductive components [205]. Functional anodes modified with CNT’s apparently achieved a desirable and improved output power [206]. As shown in Table 6, the comparative effects of nanomaterials on anode modification in MFCs was presented.

**Table 6:** Comparative effects of nanomaterials on anode modification in MFCs

Anode/cathode	Modifier	Modification technique	Efficiency	Potential applied (V)	Application	Refs.
Graphite felt	PANI and GO	Electro- polymerization	1.2A/m^2^	0.7	Bio electrochemical system	[207, 208]
Graphene	NH_4_ group	Molecularly imprinting	–	0.8	4-nonylphenol detection	[209, 210]
Stainless steel fiber felt	Metal hydroxides/oxides with 3D open nanoporous structures	Mechanical grinding/electrochemical	0.6 mA	1.1	Hydrogen production	[211, 212]
Carbon cloth	Pt/CNT	Electrochemical	0.21 m^3^H_2_/m^3^/d	1.35	–	[213]
Carbon cloth/ stainless stee	Plasma	Electrochemical	–	0.6	–	[214]
Graphite plate	Nickel oxide (NiO) and rGO	Electro- polymerization	1.16A/m_2_	1.0	Bio electrochemical system	[215]
Glassy carbon	Nanoparticles (NPs)	Immobilization	–	–	Electrochemical oxidation of naproxen	[216]

### Challenges and opportunities

MFC technology has become more and more common in recent years, although it has its number of challenges [217]. The performance of MFCs is affected by a number of factors, including the rate of fuel oxidation, circuit resistance, electrons that the microorganisms transport to the electrode, proton transfer over the membrane to the cathode, reduction at the cathode, and oxygen supply[218]. Short lifespans, low production rates, high prices, membrane fouling, limited efficiency, instability, and the hassle of maintaining microbe-based systems are some of the key disadvantages of MFCs [13]. Concerns have long existed regarding the sustainability of cathode catalysts and, frequently, membrane breakdown during the life of MFCs [219]. The utilization of MFCs is typically restricted by the exorbitant cost of materials. The incapacity of microbial fuel cell technology to generate sufficient electricity is its main drawback. Second, the expensive cost of membranes, cathode catalysts, and electrode materials hinders the technology’s advancement [220]. Using ultra-capacitors as a suitable power management system is an additional possibility because the power produced by the MFCs might not be sufficient to run a transmitter or sensor continuously [221].

### Future scopes of MFCs

To reduce the losses brought on by activation, ohmic, and concentration overpotentials, low power densities in MFC operation must be explored [222]. Optimizing the existing designs is one way to achieve this [223]. Targeting losses brought on by unnecessary chemical processes is also crucial. Examples of these include the direct oxidation of fuel by O_2_ passage into the anodic chamber or detrimental microbial metabolic activity [224]. The volumetric capacity of the system must be raised, nonetheless, without resulting in internal energy losses. Through electrode surface modification and the use of active catalyst coating, improved electron transport mechanisms between the electrode and the biocatalyst are being studied. Another crucial area that must be taken into account for the ongoing development of the MFC is mathematical modeling [225]. This helps to integrate our understanding of MFCs and their operations, pinpoint the variables influencing the generation of power, and provide guidance for scaling-up methods [226]. The primary materials used in the electrodes and membranes of MFCs must be more affordable and have a longer lifespan in order for the technology to compete with other sustainable waste-to-energy options. Mass transfer to and from the electrodes can be increased by altering the electrodes with various nanocomposite materials [227].

## Conclusion

Production of energy and treatment of wastewater are two of the most important worldwide issues that MFCs provide as a promising green technology. Through the utilization of electroactive microorganisms’ metabolic processes, MFCs potentially offer a sustainable solution for converting organic waste into bioelectricity while simultaneously purifying wastewater. The performance of MFCs is greatly influenced by the anode material selection, and the incorporation of nanomaterials such metal oxides, graphene, and CNTs has demonstrated considerable promise in improving electron transport, microbial adhesion, and overall efficiency. This review highlights the advancements in anode modifications, emphasizing how these nanoscale improvements contribute to both increased power output and improved wastewater treatment efficiency. Moreover, MFCs’ ability to reduce pollutants, such as COD and other organic contaminants, positions them as a viable alternative for sustainable energy production and environmental remediation. However, challenges remain in scaling up MFC technology for commercial applications, particularly in terms of cost, system stability, and long-term performance. Future research should focus on improving material sustainability, reducing system costs, and optimizing design configurations to enhance power generation and treatment efficiency on an industrial scale. With continued advancements in nanotechnology and bioelectrochemical systems, MFCs have the potential to play a key role in the global transition to cleaner energy and environmentally friendly wastewater treatment solutions. In addition, the creation of biofilms through genetic engineering and synthetic biology has recently drawn the interest of researchers who hope to increase the efficiency of MFCs by improving electron transfer, energy recovery, biofilm growth, and metabolic efficiency.

## Abbreviations

AC: Alternating current
Al_2_O_3_: Aluminum (III) oxide
BEC: Bioelectrochemical cell
BESs: Bioelectrochemical systems
CNTs: Carbon nanotubes
CNFs: Carbon nanofibers
CV: Cyclic voltammetry
COD: Chemical oxygen demand
CE%: Coulombic efficiency
CNs: Carbon nanospheres
DET: Direct electron transfer
DNA: Deoxyribonucleic acid
DC: Direct current
EAB: Electrochemically active bacteria
EABF: Electroactive biofilm
EAMs: Electroactive microorganisms
EET: Extracellular electron transfer
EESs: Enzymatic electrochemical systems
EIS: Electrochemical impedance spectroscopy
EPS: Extracellular polymeric substances
eDNA: Extracellular DNA
Fe_3_O_4_: Iron (II, III) oxide
Gr: Graphene
HGT: Horizontal gene transfer
IET: Indirect electron transfer
LED: Light emitting diode
MECs: Microbial electrolysis cells
MESs: Microbial electrosynthesis systems
MDCs: Microbial desalination cells
MSCs: Microbial solar cells
MFCs: Microbial fuel cells
MESs: Microbial electrochemical systems
MWCNT: Multi walled carbon nanotube
MnO_2_: Manganese oxide
NASA: National aeronautics and space administration
NA: Not available
ORR: Oxygen reduction reaction
OMVs: Outer membrane vesicles
PMFCs: Plant microbial fuel cells
Pt: Platinum
PPy: Poly-pyrrole
PEDOT: Poly (3, 4-ethylene-dioxythiophene)
PANI: Poly-aniline
PEM: Proton exchange membrane
Pmax: Performance max
QS: Quorum sensing
rGO: reduced graphene oxide
SM-CNTs: Surface-modified
TiO_2_: Titanium oxide
TEA: Terminal electron acceptor
UV: Ultraviolet
WE: Working electron
WTP: Water treatment plant
